# Malignant Pustule among Wool-Sorters

**Published:** 1888-09

**Authors:** 


### CLINICAL FEATURES IN THE HISTORY OF SEVERAL CASES OF EXTERNAL ANTHRAX (MALIGNANT PUSTULE) AMONG WOOL-SORTERS.

Under the care of Samuel Lodge, M. B., B. S., Dunelm.

CASE I.—J. W. M., æt. 26, an overlooker in the washing department for camel's hair, on August 2, 1887, consulted me for supposed barber's itch. On examining his right cheek I saw a black eschar about the size of a shilling surrounded by a raised red border, with a purplish, œdematous, and indurated areola. The base was thickened and indurated, and could be felt through the mouth to be considerably larger in extent than the black eschar. There was no fœtor. Slight glandular enlargement.

*Previous History.*—On July 29, whilst being shaved, his barber cut the top from an ordinary acne pustule. On his way next morning to work amongst the camel's hair, he once more pulled the top off the spot on his cheek. He remembered touching it several times during the morning whilst at work, as it was irritable. The same evening he felt aguish, and suffered from marked febrility. The sore had a dark red base, was angry looking, itched, and burned.

*State on first Consultation.*—Anxious look on face; skin rather dry. Temp. 100.5°. Tongue clean and moist. Pulse 90. Resp. 23. Patient was told the exact nature of the case, and its contagious power was explained. Pustule was dressed with my father's antiseptic vegetable silk, impregnated with boracic acid, and occasionally swabbed over with corrosive sublimate solution, 1–1000. Microscopically I detected the bacillus anthracis in the serum from the wound. Later I asked Dr. Hime, as Medical Officer of Health, to see him, with me, and he cultivated the bacilli most successfully.

On the 31st of October there was no trace of original lesion in cheek except healthy cicatrix.

CASE II.—Samuel G., 21, van mohair sorter. On Friday night a papule about the size of a pin's head appeared on right upper eyelid. The same evening papule became vesicular, and had a bluish areola.

Saturday morning.—Patient could hardly see, on account of œdema. Saw a chemist who told him it was erysipelas, and prescribed for him.

Sunday morning.—Pustule larger, congestion increasing, cervical glandular enlargement. Characteristic black eschar on pustule. General symptoms much worse. Head, neck, mouth, and chest on same side all swollen.

Tuesday.—Could not see out of either eye, pustules on buccal mucous membrane, saliva dribbled from mouth. Incisions made into congested tissues. Additional symptoms were slight cough and dyspnœa.

Wednesday morning.—Increasing glandular enlargement. Congestion much relieved by deep incision. In the evening, rigors, incessant vomiting, marked delirium, diarrhœa, with "meat washings" stools every ten minutes. Patient died next day.

Pustules in mouth seemed to be secondary pustules. General anthrax seemed to supervene, also complicated with intestinal anthrax (mycosis intestinalis), judging from "meat washings" stools.

CASE III.—Pustule commenced on eyebrow. Similar in character to Case II, and ended fatally.

CASE IV.—Arm accidentally injured whilst at work. Patient wore a cadaverous expression. Pulse at wrist imperceptible. Arm considerably enlarged (15 inches over biceps). From subcutaneous emphysema, due probably to decomposition of subcutaneous fat, the arm was dry and papery-like—a needle passed through skin as if into a hollow body. Cicatrix of recent wound dried up and scaly-looking. Temperature in mouth 95°. Suppression of urine. Collapsed but sensible, answering questions coherently. Supra-clavicular glandular enlargement.

*Autopsy.*—Nothing of importance beyond characteristic blueness of extremities (first described by my father in 1855).

CASE V.—Malignant pustule situated on the arm, similar in character to last, the whole limb rapidly swelling and losing heat. Man recovered finally, but with an impaired constitution.

CASE VI.—A revolving band stripped the tissues from the forearm of a young woman almost to the bone. After the wound filled with healthy granulations, a typical malignant pustule commenced on the raw edges and spread eccentrically, and before the wound was filled up the pustule had disappeared. The raw surface kept healthy all the time. It seemed worthy of notice first, that the disease never attacked the raw surface, but only spread eccentrically in the skin. Secondly, the spreading in the skin agrees with the ordinarily observed cases, and accounts for the frequency with which the diagnosis of erysipelas is at first made. The contrast between the infiltration of noma and malignant pustule is here shown to be very great.

CASE VII.—Condition of patient at first visit: Three large vesicles, not unlike herpes in character, but unlike it in distribution, as one pustule was situated on the top of the right shoulder, another over angle of left scapula, the remaining one midway between the other two. The gentleman who first saw the case remarked that "it was the queerest vesicle he had ever seen, and thought herpes resembled it most, but could not understand the vesicles being larger than a shilling, and why there should be such great œdema and constitutional symptoms." He further added that "the resemblance in the symptoms in this case to those in some cases of snake-poisoning in the Australian bush was very striking." The patient died in three days.—*Medical Press and Circular.*

